# A review of state regulations to promote infant physical activity in child care

**DOI:** 10.1186/s12966-014-0139-3

**Published:** 2014-11-22

**Authors:** Meghan M Slining, Sara E Benjamin Neelon, Kiyah J Duffey

**Affiliations:** Department of Health Sciences, Furman University, 3300 Poinsett Highway, Greenville, SC 29613 USA; Department of Community and Family Medicine, Duke University Medical Center, Durham, NC USA; Department of Human Nutrition, Foods and Exercise, Virginia Tech, Blacksburg, VA USA

**Keywords:** Child care, Infant, Obesity, Policy, Regulations, Physical activity

## Abstract

**Background:**

The purpose of this study was to review state regulations promoting increased physical activity and decreased sedentary behaviors in infants in child care and to assess consistency with recent Institute of Medicine (IOM) recommendations.

**Methods:**

We compared existing state and territory licensing and administrative regulations to recent IOM recommendations to promote physical activity and decrease sedentary time in very young children attending out-of-home child care (both child care centers and family child care homes). Three independent reviewers searched two sources (a publicly available website and WestlawNext™) and compared regulations with five IOM recommendations: 1) providing daily opportunities for infants to move, 2) engaging with infants on the ground, 3) providing daily tummy time for infants less than six months of age, 4) using cribs, car seats and high chairs for their primary purpose, and 5) limiting the use of restrictive equipment for holding infants while they are awake. We used Pearson chi-square tests to assess associations between geographic region, year of last update, and number of state regulations consistent with the IOM recommendations.

**Results:**

The mean (SD) number of regulations for states was 1.9 (1.3) for centers and 1.6 (1.2) for homes out of a possible 5.0. Two states had regulations for all five recommendations, Arizona for centers and Virginia for homes. Six states and territories had zero regulations for child care centers and seven states and territories had zero regulations for family child care homes. There were no significant associations between geographic region and number of regulations consistent with IOM recommendations.

**Conclusions:**

Out-of-home child care settings are important targets for optimal early child health interventions. While most states had some regulations related to the promotion of physical activity among infants, few states had regulations for more than three of the five IOM recommendations. Enhancing state regulations in child care facilities could aid in early childhood obesity prevention efforts.

## Background

Rates of childhood overweight and obesity continue to remain elevated in the United States (US) [1]. Of particular concern, recent estimates suggest that even among infants and toddlers rates are high; 8.1% of children from birth to aged two years in the US have excess weight for their length (at or above the 95th percentile of sex-specific Centers for Disease Control (CDC) weight for length growth charts) [1]. Excess weight among infants and toddlers is associated with a number of adverse health outcomes, including delayed gross motor development [2,3] and persistence of excess weight into childhood and adolescence [4], where evidence demonstrates further adverse health outcomes [5]. Thus, efforts to promote healthy growth and prevent obesity should begin in the first years of life.

In children both increased physical activity and decreased sedentary behaviors have been associated with lower overweight and obesity [6,7] and as such are key targets for obesity prevention efforts. Limited evidence suggests that even among infants, increased or higher physical activity is positively associated with improved measures of adiposity [8]. In toddlers, evidence suggests increased or higher physical activity is positively associated with bone and skeletal health [9] and motor skill development [10]. Further, both sedentary behaviors and physical activity appear to track consistently from early childhood to middle childhood [11].

Recently the Institute of Medicine (IOM) released policy-based recommendations to help prevent obesity in young children [12], including recommendations for state licensing and administrative agencies to increase physical activity and decrease sedentary behaviors in child care facilities. Because over 61% of children under the age of five years in the US spend time in regular out-of-home child care [13], this setting has emerged as an important target for obesity prevention [14]. While the IOM recommendations were an important step to encourage physical activity among infants for obesity prevention in child care settings, little is known about the extent to which these recommendations are being implemented by states. The purpose of this study was to review state regulations promoting increased physical activity and decreased sedentary behaviors in very young children in child care, assess consistency with the IOM recommendations, and explore geographic differences in states meeting the IOM recommendations.

## Methods

### Overview

For this cross-sectional study, we compared existing state licensing and administrative regulations to recent IOM recommendations to promote physical activity and decrease sedentary time in very young children attending out-of-home child care. Since this study was a policy review and did not involve human subjects, ethical approval was not required by Furman University.

### Physical activity recommendations

We identified two recommendations from the IOM policy report that targeted healthy physical activity and sedentary behavior practices related to obesity prevention for infants attending out-of-home child care programs. The first recommendation stated that “child care regulatory agencies should require child care providers and early childhood educators to provide infants, toddlers, and preschool children with opportunities to be physically active throughout the day”. The IOM noted three potential actions (“recommendations”) to achieve this goal in infants, including 1) providing daily opportunities for infants to move freely under adult supervision to explore their indoor and outdoor environments, 2) engaging with infants on the ground each day to optimize adult-infant interactions, and 3) providing daily “tummy time” (time in the prone position) for infants younger than six months of age. The second recommendation stated that “child care regulatory agencies should require child care providers and early childhood educators to allow infants, toddlers, and preschoolers to move freely by limiting the use of equipment that restricts infants’ movement and by implementing appropriate strategies to ensure that the amount of time toddlers and preschoolers spend sitting or standing still is limited.” The IOM noted two potential actions (“recommendations”) to achieve this goal in infants, including 1) using cribs, car seats, and high chairs for their primary purpose only—cribs for sleeping, car seats for vehicle travel, and high chairs for eating, and 2) limiting the use of equipment such as strollers, swings, and bouncer seats/chairs for holding infants while they are awake [12]. Thus, we were interested in these five recommendations related to promoting physical activity and limiting sedentary time in infants in child care.

### State regulations review

We reviewed each state’s licensing and administrative regulations for child care facilities between August and December of 2013, focusing on regulations consistent with the five IOM recommendations. We searched two sources for regulations using primary legal research methods: a publically available website maintained by the National Resource Center for Health and Safety in Child Care in partnership with the American Academy of Pediatrics (www.nrckids.org) and the commercial legal research database WestlawNext™. Each state’s regulations were coded by a trained reviewer (first author) using a combination of Boolean key word searches and review of the full text, consistent with previous policy reviews [15]. We reviewed regulations for all 50 US states, the District of Columbia (DC), Puerto Rico, the US Virgin Islands (USVI), Guam, and the Department of Defense. The Department of Defense child care regulations govern facilities in residential areas for US soldiers and their dependents stationed both domestically and abroad. We documented regulations consistent with each of the five IOM actions for healthy physical activity and sedentary behavior practices in child care [12]. To be counted, regulations needed to include clear and specific language embodying the spirit of the IOM recommended actions. We also recorded the date of the most recent regulation update to evaluate if the regulation was adopted prior to or after the release of the IOM recommendations.

We reviewed regulations for both child care centers (“centers”) and family child care homes (“homes”). Generally, centers care for more children at once, have a number of staff members, and are located in a dedicated building that is not a home. Homes, on the other hand, typically include a single care provider who is often the home owner, and care for fewer children—often of mixed ages. Some states regulate different a number of types of centers and homes (e.g., infant care centers, large family child care homes). Where appropriate, we grouped these types of facilities into either “centers” or “homes” for the purpose of reporting results of this review. For example, we classified infant care centers as centers and large family child care homes as homes.

### Analysis

We computed means, frequencies, and standard deviations (SD) for the number of regulations for each state consistent with the five IOM recommendations, according to type of facility and geographic census region (Northeast, South, Midwest, and West). We used Pearson chi-square tests to assess associations between the geographic region of the state and the number of regulations consistent with IOM recommendations. Next, we computed Spearman correlation coefficients to examine the relationship between the year the regulation was last updated, treated as a continuous variable, and the number of regulations in each state, treated as an ordinal variable ranging from 0 to five. Additionally, we used Pearson chi-square tests to explore associations between the dichotomized year variable (prior to the release of the IOM recommendations versus after the release) and number of regulations in each state. We conducted all analyses using Stata version 13.1 (College Station, Texas, US), with a significance level of α = 0.05.

## Results

Overall, most states had some regulations related to the promotion of physical activity among infants (Table 1). The mean (SD) number of regulations for states was 1.9 (1.3) for centers and 1.6 (1.2) for homes out of a possible 5.0. Two states had regulations for all five recommendations, Arizona for centers and Virginia for homes. Delaware had regulations for four of the five recommendations in both centers and homes. Two additional states, Connecticut and Washington, had regulations for four of the five recommendations for centers. Six states and territories had zero regulations for child care centers and seven states and territories had zero regulations for family child care homes.

**Table 1:** **State regulations for child care centers and family homes consistent with IOM infant physical activity recommendations**

**State**	**Facility type**	**Year of last update**	**Provide daily opportunities for infants to move freely**	**Engage with infants on the ground**	**Provide daily tummy time for infants less than 6 months**	**Use cribs, car seats, and high chairs for their primary purpose**	**Limit use of restrictive equipment for holding infants while awake**	**Total**
AL	Centers	2007	X		X			2
	Homes	2007			X			1
AK	Centers	2007	X			X	X	3
	Homes	2007	X			X	X	3
AZ	Centers	2010	X	X	X	X	X	5
	Homes	2011				X	X	2
AR	Centers	2011	X			X	X	3
	Homes	2011				X		1
CA	Centers	2008	X					1
	Homes	2009						0
CO	Centers	2012	X			X	X	3
	Homes	2012	X			X	X	3
CT	Centers	2013	X		X	X	X	4
	Homes	2013	X		X	X		3
DE	Centers	2007	X		X	X	X	4
	Homes	2009	X	X	X		X	4
FL	Centers	2010	X					1
	Homes	2010	X					1
GA	Centers	2013	X				X	2
	Homes	2012	X			X	X	3
HI	Centers	2002						0
	Homes	2002						0
ID	Centers	2011						0
	Homes	2011						0
IL	Centers	2010	X		X			2
	Homes	2010			X			1
IN	Centers	2003	X			X	X	3
	Homes	2001	X					1
IA	Centers	2012						0
	Homes	2012						0
KS	Centers	2012				X	X	2
	Homes	2012						0
KY	Centers	2008						0
	Homes	2008						0
LA	Centers	2012				X	X	2
	Homes	--					X	1
ME	Centers	2008						0
	Homes	2009	X					1
MD	Centers	2012	X					1
	Homes	2012	X					1
MA	Centers	2010	X				X	2
	Homes	2010	X				X	2
MI	Centers	2008				X		1
	Homes	2009				X		1
MN	Centers	2010						0
	Homes	2007	X					1
MS	Centers	2009	X					1
	Homes	2009	X					1
MO	Centers	2011	X			X	X	3
	Homes	2011	X			X	X	3
MT	Centers	2012	X			X	X	3
	Homes	2012	X			X	X	3
NE	Centers	2013						0
	Homes	2013						0
NV	Centers	2012	X			X	X	3
	Homes	2012	X			X	X	3
NH	Centers	2008					X	1
	Homes	2008					X	1
NJ	Centers	2009	X				X	2
	Homes	2009					X	1
NM	Centers	2012				X	X	2
	Homes	2012				X	X	2
NY	Centers	2005	X			X	X	3
	Homes	2005	X			X	X	3
NC	Centers	2013	X		X			2
	Homes	2013			X			1
ND	Centers	2013	X					1
	Homes	2013	X					1
OH	Centers	2010	X			X	X	3
	Homes	2011				X	X	2
OK	Centers	2010	X			X	X	3
	Homes	2010					X	1
OR	Centers	2011	X			X	X	3
	Homes	2011	X			X	X	3
PA	Centers	2009						0
	Homes	2009						0
RI	Centers	1993	X			X		2
	Homes	2007						0
SC	Centers	2005				X	X	2
	Homes	2005				X	X	2
SD	Centers	2013						0
	Homes	2013	X					1
TN	Centers	2009	X			X	X	3
	Homes	2009						0
TX	Centers	2013	X		X		X	3
	Homes	2013	X		X		X	3
UT	Centers	2013	X			X	X	3
	Homes	2013	X			X	X	3
VT	Centers	2001	X				X	2
	Homes	2001	X				X	2
VA	Centers	2012	X		X			2
	Homes	2013	X	X	X	X	X	5
WA	Centers	2013	X		X	X	X	4
	Homes	2013			X	X		2
WV	Centers	2009	X			X	X	3
	Homes	2012	X			X	X	3
WI	Centers	2009	X		X			2
	Homes	2009	X		X			2
WY	Centers	2013	X				X	2
	Homes	2013	X				X	2
PR	Centers	1992						0
	Homes	1992						0
USVI	Centers	2011	X			X	X	3
	Homes	2011	X			X	X	3
GU	Centers	1997						0
	Homes	1997						0
DC	Centers	2007	X		X			2
	Homes	2007			X	X		2
DOD	Centers	1996						0
	Homes	1996						0

PR, Puerto Rico; USVI, United States Virgin Islands; DOD, Department of Defense.

Thirty-seven states (including DC and USVI) had regulations for centers and 27 had regulations for homes consistent with the recommendation to provide daily opportunities for infants to move freely. The second most common regulation was related to limiting the use of restrictive equipment for holding infants while awake (37 states had center regulations and 25 had home regulations consistent with that recommendation). Relatedly, the third most common regulation was using cribs, car seats and high chairs for their primary purposes. Twenty-five states had center regulations and 21 had home regulations consistent with that recommendation. Less than 20% of states had regulations consistent with the recommendation to provide daily tummy time for infants younger than 6 months. Eleven states had center regulations and 10 states had home regulations consistent with that recommendation. Arizona, Delaware and Virginia were the only states with a regulation that caregivers engage with infants on the ground, Arizona with a center regulation and Delaware and Virginia with home regulations.

When we examined geographic differences, we found that states in the West had the greatest mean (SD) number of regulations for centers [2.5 (1.5)] and homes [1.1 (0.6)], compared with the Midwest, which had the fewest for centers [1.4 (1.2)] and homes [1.1 (0.9)]. These associations between geographic region and number of regulations were not significant for either centers (p = 0.85) or homes (p = 0.42). Approximately two thirds of states had not updated their regulations in recent years. However, nineteen states had updated their regulations in 2012 or 2013—after the IOM recommendations were released.

The number of regulations consistent with IOM recommendations examined as a dichotomized variable (before the recommendations were released vs. after) was not associated with the year of last update for centers (p = 0.85), nor homes (p = 0.07). In contrast, while the number of regulations was not correlated with the year of last update examined as a continuous variable for centers (Spearman’s rho = 0.15; p = 0.28), it was positively correlated for homes (Spearman’s rho = 0.30; p = 0.03).

## Discussion

In this review of US state regulations for child care facilities we found considerable variation among and within states regarding regulations related to the promotion of physical activity among infants in child care settings. While most states had some regulations related to the promotion of physical activity among infants, few states had regulations for more than three of the five Institute of Medicine recommendations. For child care centers, 12 states (22%) did not have any regulations related to the five physical activity recommendations and only four states (7%) had four or five of the five recommendations. For family child care homes, 13 states (24%) did not have any regulations related to the five physical activity recommendations and only 2 states (4%) had four or five of the five recommendations. Most states had between one and three regulations related to the IOM recommendations for infant physical activity. We did not find statistically significant associations between geographic region and number of regulations but did find a positive correlation between the year of last update for family child care homes and the number of regulations consistent with the IOM recommendations.

To our knowledge this is the first study to examine state regulations related to the promotion of physical activity exclusively among infants in child care settings. Our findings are similar to those previously reported for children in child care settings [14,16-18]. Similar to our findings in infants, physical activity regulations for children in child care settings varied markedly among and within states [16,18]. Further, comparable previous reviews also found few regulations related to physical activity promotion in child care settings among children of all ages [17,18].

The importance of movement in early life is increasingly recognized. Evidence showing that physical activity in infancy is related to body fatness in both infancy [19] and later childhood [8] and that infant fatness influences both later fatness and activity patterns [20] has established the infancy period as an important target of childhood obesity prevention. Moreover the health benefits of movement in early life extend beyond obesity prevention. Infant motor activity may also play a significant role in determining developmental sequences in other domains, including social, perceptual and cognitive development [21-23].

The most common state regulations were to provide daily opportunities for infants to move freely and to limit the use of restrictive equipment for holding infants while awake. Caregivers can further facilitate physical activity in infants by engaging with them on the floor, as highlighted in the IOM report [12]. Only three states (Arizona, Delaware and Virginia) had regulations that caregivers engage with infants on the ground. Evidence also suggests that infants should spend time daily in the prone position (“tummy time”) to improve motor milestone achievement [24], though less than 20% of states had regulations consistent with the IOM recommendation for daily tummy time for infants.

Additional national efforts to develop policies related to nutrition and physical activity acknowledge the importance of the childcare setting for obesity prevention. Let’s Move! Childcare has identified five goals for obesity prevention in childcare settings related to physical activity, screen time, food, beverages, and infant feeding [25]. Specific recommendations provided for infant physical activity (keeping infants active daily, minimizing time in restrictive baby equipment, and providing daily tummy time) are consistent with the IOM recommendations [25]. Furthermore, efforts are underway to include recommendations specific to infants in the 2020 Dietary Guidelines for Americans [26]. Previously released guidelines have emphasized the role of physical activity in achieving calorie balance [27].

One strength of this study is the separate evaluation of child care centers and family child care homes as these settings are distinct. The number of children and providers as well as the physical environments of centers and family child care homes differ. Previous regulatory reviews have shown that state regulations differ for centers and family child care homes [16-18], with centers generally more heavily regulated and containing more specific regulations [16]. Finally, previous research has suggested that child care in someone else’s home during the first six months of life is associated with subsequent increased weight gain [28].

It is important to note some limitations to this study. First, these types of policy reviews become outdated quickly, as many states are in the process of updating their regulations at any given time. Thus, this review is current as of 2013, but states may have revised their regulations since that time. Next, this review describes the presence of state regulations, but does not examine actual practices within child care settings. Although child care facilities are required by law to adhere to their state regulations, this does not necessarily translate into regular practice. Penalties associated with not adhering to regulations vary by state, but typically include a written warning to comply and a possible fine for continued non-compliance. Further, the lack of a state regulation does not confirm that the practice is not in effect in the state or territory. Finally, it is possible that regulations exist but were missed in our review.

## Conclusion

Efforts to promote healthy growth and prevent obesity should begin in the first year of life. Out-of-home child care settings are important targets for optimal early child health interventions. A recent IOM report including policy recommendations for state licensing and administrative agencies aimed at increasing physical activity and decreasing sedentary behaviors in child care facilities. We found that while most states had some regulations related to the promotion of physical activity among infants, few states had regulations for more than three of the five IOM recommendations. Enhancing and enforcing state regulations in child care facilities could aid in early childhood obesity prevention efforts.

## Abbreviations

CDC: Centers for Disease Control
DC: District of Columbia
DOD: Department of Defense
IOM: Institute of Medicine
SD: Standard deviation
US: United States
USVI: United States Virgin Islands

## References

[1] Ogden CL, Carroll MD, Kit BK, Flegal KM (2014). Prevalence of childhood and adult obesity in the United States, 2011–2012. JAMA.

[2] Slining M, Adair LS, Goldman BD, Borja JB, Bentley M (2010). Infant overweight is associated with delayed motor development. J Pediatr.

[3] Benjamin Neelon SE, Oken E, Taveras EM, Rifas-Shiman SL, Gillman MW (2012). Age of achievement of gross motor milestones in infancy and adiposity at age 3 years. Matern Child Health J.

[4] Mei Z, Grummer-Strawn LM, Scanlon KS (2003). Does overweight in infancy persist through the preschool years? An analysis of CDC pediatric nutrition surveillance system data. Soz Praventivmed.

[5] Must A, Jacques PF, Dallal GE, Bajema CJ, Dietz WH (1992). Long-term morbidity and mortality of overweight adolescents. A follow-up of the Harvard Growth Study of 1922 to 1935. N Engl J Med.

[6] Janssen I, Leblanc AG (2010). Systematic review of the health benefits of physical activity and fitness in school-aged children and youth. Int J Behav Nutr Phys Act.

[7] Strong WB, Malina RM, Blimkie CJ, Daniels SR, Dishman RK, Gutin B, Hergenroeder AC, Must A, Nixon PA, Pivarnik JM, Rowland T, Trost S, Trudeau F (2005). Evidence based physical activity for school-age youth. J Pediatr.

[8] Wells JC, Ritz P (2001). Physical activity at 9–12 months and fatness at 2 years of age. Am J Hum Biol.

[9] Specker B, Binkley T (2003). Randomized trial of physical activity and calcium supplementation on bone mineral content in 3- to 5-year-old children. J Bone Miner Res.

[10] Jones RA, Riethmuller A, Hesketh K, Trezise J, Batterham M, Okely AD (2011). Promoting fundamental movement skill development and physical activity in early childhood settings: a cluster randomized controlled trial. Pediatr Exerc Sci.

[11] Jones RA, Hinkley T, Okely AD, Salmon J (2013). Tracking physical activity and sedentary behavior in childhood: a systematic review. Am J Prev Med.

[12] Institute of Medicine (2011). Early Childhood Obesity Prevention Policies.

[13] **Who’s Minding the Kids? Child Care Arrangements: Spring 2011.** [http://www.census.gov/prod/2013pubs/p70-135.pdf]

[14] Larson N, Ward DS, Neelon SB, Story M (2011). What role can child-care settings play in obesity prevention? A review of the evidence and call for research efforts. J Am Diet Assoc.

[15] Mersky RM, Dunn DJ (2002). Fundamentals of Legal Research.

[16] Kaphingst KM, Story M (2009). Child care as an untapped setting for obesity prevention: state child care licensing regulations related to nutrition, physical activity, and media use for preschool-aged children in the United States. Prev Chronic Dis.

[17] Cradock AL, O’Donnell EM, Benjamin SE, Walker E, Slining M (2010). A review of state regulations to promote physical activity and safety on playgrounds in child care centers and family child care homes. J Phys Act Health.

[18] Benjamin SE, Cradock A, Walker EM, Slining M, Gillman MW (2008). Obesity prevention in child care: a review of U.S. state regulations. BMC Public Health.

[19] Li R, O’Connor L, Buckley D, Specker B (1995). Relation of activity levels to body fat in infants 6 to 12 months of age. J Pediatr.

[20] Wells JC, Stanley M, Laidlaw AS, Day JM, Davies PS (1996). The relationship between components of infant energy expenditure and childhood body fatness. Int J Obes Relat Metab Disord.

[21] Bushnell EW, Boudreau JP (1993). Motor development and the mind: the potential role of motor abilities as a determinant of aspects of perceptual development. Child Dev.

[22] Piek JP, Dawson L, Smith LM, Gasson N (2008). The role of early fine and gross motor development on later motor and cognitive ability. Hum Mov Sci.

[23] Miquelote AF, Santos DCC, Caçola PM, Montebelo MIDL, Gabbard C (2012). Effect of the home environment on motor and cognitive behavior of infants. Infant Behav Dev.

[24] Salls JS, Silverman LN, Gatty CM (2002). The relationship of infant sleep and play positioning to motor milestone achievement. Am J Occup Ther.

[25] **Let’s Move! Child Care.** [https://www.healthykidshealthyfuture.org/home/startearly/thegoal.html]

[26] **Questions and Answers on the 2015 Dietary Guidelines for Americans.** [http://www.health.gov/dietaryguidelines/2015.asp#GO5]

[27] USDA, USDHHS (2010). Dietary Guidelines for Americans.

[28] Benjamin SE, Rifas-Shiman SL, Taveras EM, Haines J, Finkelstein J, Kleinman K, Gillman MW (2009). Early child care and adiposity at ages 1 and 3 years. Pediatrics.

